# Research the Dimensional Accuracy of C45 Steel Ring Forgings Produced by Radial Rolling

**DOI:** 10.3390/ma17010003

**Published:** 2023-12-19

**Authors:** Andrzej Gontarz, Piotr Surdacki, Jacek Michalczyk

**Affiliations:** 1 Faculty of Mechanical Engineering, Lublin University of Technology, 20-618 Lublin, Poland; piotr.surdacki@pollub.pl; 2 Faculty of Production Engineering and Materials Technology, Czestochowa University of Technology, 42-201 Czestochowa, Poland; michalczyk.jacek@wip.pcz.pl

**Keywords:** radial ring rolling, steel C45, technological parameters, dimensional accuracy

## Abstract

The rolling process of rings is a commonly used method for producing annular forgings. There are two primary types of this process: radial-axial rolling and radial rolling. This article presents the research results regarding the latter, in which obtaining a product with the assumed dimensions constitutes a major problem. In industrial practice, the process parameters are based on the experience of technologists and/or by trial and error. This is why the authors considered it justified to undertake the research aimed at determining the influence of the main process parameters, that is, preform temperature and tool speed, on the shape and dimensions of the cross-section, which determine the internal and external diameters of the rolled ring. The research was based on numerical simulations and experimental studies. The results obtained proved that the higher the feed speed of the main roll, the greater the change in the cross-sectional height during rolling, and the smaller the cross-sectional deformation (the so-called fishtail). Nevertheless, a higher preform temperature reduces the final height of the ring and reduces cross-section deformation. On the basis of the obtained test results, guidelines for the process design were postulated, considering the influence of temperature and speed parameters on the final dimensions of the forging and the dimensions of the preform.

## 1. Introduction

The development of machines and devices is related to the industry’s demand for the annular elements. An efficient technique of manufacturing this type of part is the production of forged rolled rings in which the main operations include forging a preform—the preliminary ring, rolling the ring forging, and the mechanical processing. The main stage of this process is rolling which allows the formation of rings with large diameters in relation to the cross-section dimensions, uniform structure, small machining allowances, and a large array of dimensions while maintaining the efficiency unachievable with other manufacturing methods.

**Figure 1:**
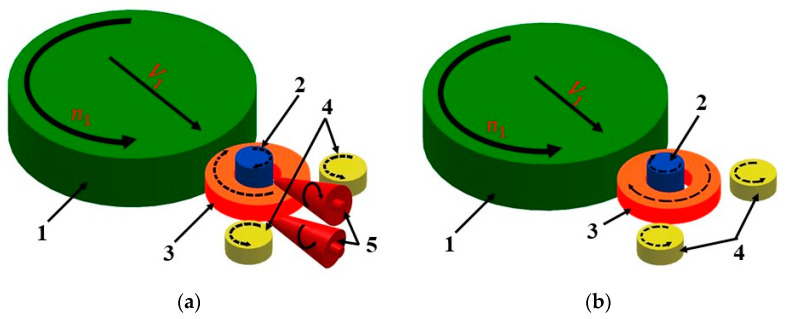
Diagram of the rolling method: (**a**) radial-axial, (**b**) radial; 1—main roll, 2—mandrel, 3—rolled ring, 4—guide rolls, and 5—conical rolls.

The ring rolling technology has been used and developed for over 150 years, and significant research work has been carried out in this area over the last 50 years. Two leading methods are used: radial-axial rolling and radial rolling (Figure 1) [1,2,3,4].

Radial-axial rolling of rings (Figure 1a) involves reducing the cross-section in the radial direction of the product. In such a process, it is possible to roll the preform axially using the conical rolls mainly to reduce the height of the ring. The use of the conical rolls allows for controlling the height of the ring. This method is most often used when shaping large rings and is performed in a horizontal arrangement.

Radial rolling, the subject of this publication, involves reducing the cross-section of the product only in the radial direction. The rolled ring is placed on the mandrel (Figure 1b). The reduction of the cross-section of the ring is a result of the infeed at the *V*_1_ speed of the main roll (in some rolling mills, the infeed is performed by the mandrel). The main roll also rotates at a rotational speed *n*_1_, causing the ring and mandrel to continuously rotate around their own axes. The guide rolls are also involved in the process to calibrate the ring and provide support points for the rolled semi-product. In some machines, these rolls can move as the diameter of the product increases, providing a continuous point of support.

More often, however, they do not have the functionality of continuous movement, one of the rolls provides a support point at a certain stage of the process while the other provides a support point and allows for the final calibration of the ring at the final stage.

The ring rolling process is mainly carried out hot, therefore, this method can produce rings from all metals and alloys showing good plasticity at temperatures higher than the recrystallization temperature. Production plants sometimes use cold rolling in the final phase of production. The most commonly cold rolled ring is a ring previously obtained by hot rolling, usually without a significant increase in its diameter. The purpose of cold rolling is to improve surface quality and dimensional accuracy as well as to increase strength and fatigue strength [5].

The basic condition for ring rolling is that the preform is continuously drawn into the gap where it is forming. If the thickness is reduced too much, depending on the speed and dimensional parameters of the tools, slippage occurs and rolling cannot be performed properly. The authors of the study [6] proposed the following relationship to calculate the maximum tool feed speed:

(1) $$V_{max}=\frac{2{\cdot \theta }^{2}{\cdot n}_{1}{\cdot R}_{r}^{2}}{{R\cdot \left(1+\frac{R_{r}}{R_{m}}\right)}^{2}}\cdot \left(1+\frac{R_{r}}{R_{m}}+\frac{R_{r}}{R}-\frac{R_{r}}{r}\right),$$

where:

*n*_1_—rotational speed of the main roll,

*R_r_*—radius of the main roll,

*R*—instantaneous external radius of the shaped ring,

*r*—instantaneous internal radius of the shaped ring,

*θ*—friction angle between the rollers and the ring; *θ* = tg^−1^*µ*, where *µ* is the friction coefficient.

For speeds greater than *V_max_*, slippage occurs. However, calculating the speed *V_max_* is not easy because the problem of calculating the instantaneous value of the outer radius *R* and inner radius *r* of the ring is not fully solved.

The advantage of the radial rolling method is the use of simple tools and easier process control. The main disadvantage on the other hand is the lack of control over the height of the forging which changes (increases) during the rolling process due to the axial flow of the material. The volume of the material moving circumferentially depends on the share of the volume of the material which moves axially, which, in turn, determines the diameter of the ring. The only dimension controlled is the ring thickness, determined by the final distance between the working surface of the mandrel and the main roll which can be set by the machine operator.

The above conditions make it very difficult to control the final dimensions of the ring forging in the radial rolling process. Therefore, determining the correlation between the process parameters and the dimensions of the ring is significant as it allows for designing a process which enables obtaining forgings with the assumed dimensions and allows for reducing the allowances for mechanical processing.

**Figure 2:**
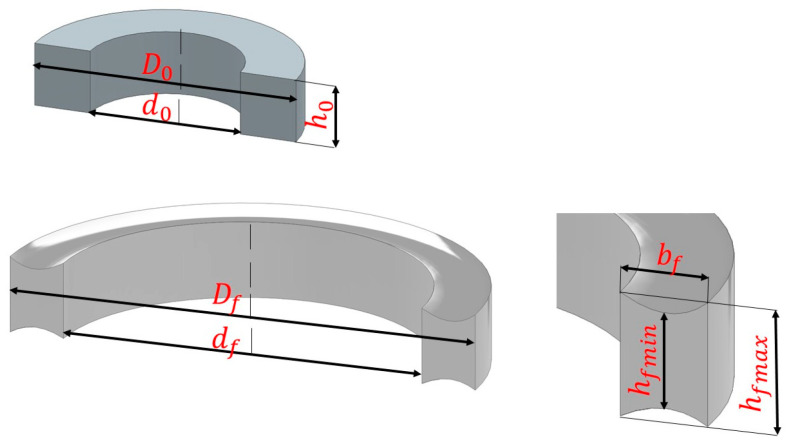
Diagram of the cross-section of the ring before and after the rolling process; *h*_0_, *D_0_*, *d_0_*—initial height, outer diameter and internal diameter of the preform, *D_f_*, *d_f_*, *b_f_*—final outer diameter, internal diameter and thickness of the ring, *h_fmax_*—final maximum height of the ring and *h_fmin_*—final minimum height of the ring.

An additional problem occurring in the radial rolling is the deformation of the cross-section of the rolled ring. This is most often an indentation of the side surfaces, the so-called “fishtail”, which results from the heterogeneity of material flow at the surface of the tools and in the middle zone (Figure 2). In publications, this deformation is most often assessed on the basis of the so-called fishtail coefficient described by the formula [7,8,9,10]:

(2) $$F=\frac{h_{fmax}-h_{fmin}}{h_{0}},$$

where:

*h_fmax_*—the maximum of axial height of a rolled ring,

*h_fmin_*—the minimum of axial height of a rolled ring,

*h*_0_—axial height of a preform.

This research presents results on the influence of the radial rolling process parameters on the phenomena mentioned above which determine the shape and dimensions of the rolled rings, specifically the change in the height of the forging during rolling and the cross-sectional deformations (the so-called fishtail) in the radial rolling process.

A review of the specialized literature showed that the vast majority of publications concern the radial-axial rolling method (for instance, recent publications [11,12,13,14,15,16]), while there are relatively few studies on the radial rolling process. Among them, there is a limited number of publications presenting the research results on the influence of the process parameters on the cross-section deformation and the final ring height (which determines the ring diameter) in the radial rolling process.

These studies mainly analyze the influence of the rotational speed and/or feed speed of the tools on the final height of the ring and on the distortion of the cross-section, measured by the fishtail coefficient determined by Equation (2).

Based on the available research results, it can be concluded that there are discrepancies in determining the influence of the rotational speed of the main roll or mandrel on the shape and cross-sectional dimensions of the rolled ring. In the study [7], the authors, examining the hot rolling process of the AZ31 magnesium alloy, reported that at a constant mandrel feed speed of 2 mm/s, the cross-sectional distortion (fishtail) decreases with an increase in the rotational speed from 20 to approximately 45 rpm, and for rotational speeds higher than 45 rpm, the defect increases. In turn, Yang H. et al. [8] in their research of cold rolling of the HE30 aluminum alloy found that the ring cross-section distortion increases with the increase in rotational speed. Other results are presented in the study [9], which describes the results of testing the hot rolling process of the Ti-64 titanium alloy. The authors found that the rotational speed of the main roll does not significantly affect the cross-sectional distortions measured by the fishtail coefficient. In the study [17] on the rolling process of AISI 1045 steel, it is stated that the fishtail effect is smaller if a variable rotational speed of the tool (main roll or mandrel) is used, ensuring a constant rotational speed of the ring during the rolling process. As you can see, the presented research on the influence of tool rotational speed on the distortion of the cross-section of the rolled ring shows significant differences. It should be mentioned that all the presented research results were obtained on the basis of numerical simulations. They have not been experimentally verified.

Another important parameter influencing the shape and dimensions of the ring is the feed speed of the main roll or mandrel (depending on the design of the rolling mill). The authors of the study [8] presented the results of theoretical tests, which show that the fishtail coefficient described by Equation (2) is greater (i.e., the cross-sectional distortion is greater) than the lower the mandrel feed speed. Similar results were presented in [9], in which, mainly based on numerical simulations, it was found that an increase in the feed speed of the main roll causes a decrease in the fishtail coefficient. In turn, analyzing the results of experimental tests presented in study [18], in which the hot rolling process of a C45 steel ring was examined, it can be concluded that an increase in the feed speed of the main roll in the range of 0.082–0.33 m/s causes a decrease in the final height of the ring, while a further increase in this speed in the range of 0.33–0.60 m/s results in an increase in the final height of the ring.

The authors of work [10] examined the Influence of the dimensions of the main roll and mandrel on the distortion of the ring cross-section. Based on numerical simulations of the hot rolling process of a ring made of titanium alloy Ti-6Al-4V, they found that the larger the radius of the main roll, the greater the fishtail coefficient, while it is possible to determine the optimal radius of the mandrel, which ensures the lowest value of this coefficient. The results of numerical simulations have not been verified experimentally.

Summarizing the analysis of the specialist literature, it ought to be stated that the vast majority of studies concern the radial-axial rolling process. Very little research has been found regarding radial rolling results. In the case of the fishtail deformation, virtually all results are obtained on the basis of numerical simulations without experimental verification. Individual works display the differences in the obtained connections between speed parameters and cross-section deformation. Even fewer studies were found describing the influence of the process parameters on the final dimensions of the ring. The authors considered it justified to fill this gap.

The analysis of the specialist publications and preliminary own research [19] revealed that the speed parameters of the process influence the final dimensions and the deformation of the cross-section of the rings. Additionally, the authors found that a significant parameter in this respect is the temperature of the shaped material. Therefore, the aim of the research was to establish the influence of the speed parameters of the radial rolling process and the preform temperature on the height and diameter of the rings as well as the deformation of the cross-section of the rolled forging (the so-called fishtail).

## 2. Materials and Methods

The tests involved the radial rolling process of rings (Figure 1b) made of 1.0503 (C45) steel, the chemical composition of which is listed in Table 1. This type of steel, easily mechanically and thermally treatable, is frequently used for the production of medium-loaded and abrasion-resistant machine parts (including those in the shape of rings). The research was carried out using a theoretical method using numerical simulations based on the finite element method and an experimental method.

Two phenomena occurring during rolling were analyzed: the height change and ring cross-section deformation. The widening coefficient, regularly used in rolling processes, was adopted as a measure of the height change, defined as:

(3) $$\alpha =\frac{h_{fa}-h_{0}}{h_{0}}\cdot \left(100\%\right),$$

where:

*h*_0_—initial height of a preform, 

*h_fa_*—average height of the cross-section after rolling (Figure 2). 

Since the height of the cross-section is not constant (the forging has a non-rectangular cross-section), and it is not possible to directly measure this dimension, the average height was used in Formula (3), expressed as:

(4) $$h_{fa}=\frac{4\cdot V_{f}}{\pi \cdot (D_{f}^{2}-d_{f}^{2})},$$

where: 

*V_f_*—ring volume after rolling,

*D_f_*—outer diameter of the ring after rolling,

*d_f_*—internal diameter of the ring after rolling.

For the quantitative assessment of the cross-sectional defect, the “fishtail” coefficient was adopted, defined slightly differently than that described in the Formula (2) and used in the literature. This coefficient was defined by relating the difference between the maximum *h_fmax_* and minimum *h_fmin_* height of the cross-section to the average height *h_fa_* of the cross-section of the finished ring and described by the relationship:

(5) $$\beta =\frac{h_{fmax}-h_{fmin}}{h_{fa}}\cdot (100\;\%),$$

where: 

*h_fmax_*—maximum cross-sectional height of the final product,

*h_fmin_*—minimum cross-sectional height of the final product,

*h_fa_*—average cross-sectional height of the final product.

It is the authors’ conclusion that the fishtail coefficient defined in this way is more objective as it refers only to the dimensions of the final cross-section, that is it expresses the discrepancy in the height of the ring cross-section in relation to the height of the ideal cross-section (rectangular, without deformations). This coefficient is independent of the initial dimensions of the preform and the degree of cross-section reduction. Therefore, it seems more universal because it can be used to compare rolling cases which differ in the geometric parameters of the process.

For the case where the final cross-section is rectangular, the *β* coefficient is equal to 0. A higher value of the *β* coefficient means a greater distortion of the cross-section in the form of the so-called fishtail. 

The speed parameters in the radial rolling process are the *V*_1_ feed speed of the main roll and the *V_t_* peripheral speed of the main roll (speed on the contact surface of the roll and the ring), which depends on the *n*_1_ rotational speed and the *D_r_* diameter of the main roll and is described by the relationship:

(6) $$V_{t}=\frac{\pi D_{r}n_{1}}{60}\;,$$

where:

*D_r_*—main roller diameter, mm,

*n*_1_—rotational speed of the main roll, rpm.

Based on the authors’ previous research on the slip phenomenon [20], it was discovered that the relationship between the mentioned speeds is very important for the correct course of the process, therefore, the coefficient *k* described by the relationship was also used when analyzing the results:

(7) $$k=\frac{V_{1}}{V_{t}}.$$

Experimental tests were carried out on D51Y–160E type commercial rolling mill, in which the rotation and feed motion is performed by the main roll. The tests used a constant rotational speed of the main roll equal to *n*_1_ = 60 rpm and a variable feed speed of the main roll in the range *V*_1_ = 0–45 mm/s. Smooth tools (cylinder-shaped) were used to roll preforms measuring ø100 × ø50 × 20 mm. The rolling continued until the rolled ring reached a thickness of *b_f_* = 13 mm. An electric resistance furnace was used to heat the preforms. In order to determine the influence of temperature on the studied phenomena, the preform was heated to temperatures within the hot forming range of the tested steel, that is, to 900 °C, 1000 °C, 1100 °C, and 1200 °C.

The theoretical analysis was carried out using the Forge NxT 1.1 program. The initial conditions were assumed to be those in the experimental tests. It was assumed that the preform was heated to the appropriate temperature. The simulation included two stages of preform cooling, corresponding to the transfer of the preform from the furnace to the rolling mill lasting *t*_1_ = 3 s and positioning the preform on the mandrel during *t*_2_ = 2 s. The three-dimensional state of strain (3D) was used in the calculations. The preform was discretized using four-node tetrahedral elements. Heat exchange between the shaped material and tools and the environment, heat generation caused by friction, and the conversion of plastic deformation work were considered. The coefficient of heat exchange between the preform and the tools was set to 10 000 W/m^2^K. The heat–air exchange coefficient was set to 20 W/m^2^K [21]. The tools were assumed as heat-conducting rigid bodies. 

The value of the friction factor was determined using the experimental and theoretical methods. Experimental tests of the rolling process were carried out for the rotational speed of the main roll *n*_1_ = 60 rpm and a temperature of 1100 °C. In each test, the feed speed *V*_1_ of the main roll was increased (in steps of 5 mm/s) up to the limiting speed at which slippage occurred. Then, maintaining the same conditions as in the experiment, sequential simulations were performed for the limiting speed (slip) and for a speed 5 mm/s lower than the limit (no slip), changing the value of the friction factor. The appropriate value of the friction factor was considered to be the one at which the simulation results were consistent with the experimental results, i.e., slippage occurred at the limiting speed, and no slippage occurred at a speed lower by 5 mm/s. Based on the presented procedure, the friction factor *m* = 0.74 was determined as a factor ensuring the best representation of real conditions in the theoretical model. 

In the analysis of hot forming processes, the deformations are relatively large and, therefore, the elastic behavior of the material is neglected. In the Forge NxT program, it is possible to use several thermo-viscoplastic material models to analyze the hot forming processes of steel. One of them that was used to describe the behavior of C45 steel is the Hansel–Spittel model described by the relationship [22]:

(8) $$\sigma =A\cdot \exp\left(m_{1}\cdot T\right)\cdot \varepsilon^{m_{2}}\cdot {\dot{\varepsilon }}^{m_{3}}\cdot \exp\left(\frac{m_{4}}{\varepsilon }\right)\cdot {\left(1+\varepsilon \right)}^{m_{5}\cdot T}{\cdot exp}^{m_{7}\cdot \varepsilon }\cdot {\dot{\varepsilon }}^{m_{8}\cdot T}\cdot T^{m_{9}},$$

where:

*σ*—flow stress (MPa),

*ε*—strain,

$\dot{\varepsilon }$—strain rate,

*T*—temperature given in degrees Celsius,

*A*—material constant,

*m*_1_*–m*_9_—model coefficients.

The coefficients *m*_1_ and *m*_9_ define the material’s sensitivity to temperature, *m*_5_ defines the coupling of temperature and strain, *m*_8_ defines the coupling of temperature and strain rate, and coefficients *m*_2_, *m*_4,_ and *m*_7_ define the sensitivity of the material to strain, while *m*_3_ depends on the material’s sensitivity to strain rate. This model takes into account the influence of temperature and strain rate as well as the phenomenon of strain hardening or strain softening.

For many materials, the value of the coefficients *m*_5_–*m*_9_ is often assumed to be zero. Then the Hansel–Spittel model takes a simplified form:

(9) $$\sigma =A\cdot \exp\left(m_{1}\cdot T\right)\cdot \varepsilon^{m_{2}}\cdot {\dot{\varepsilon }}^{m_{3}}\cdot \exp\left(\frac{m_{4}}{\varepsilon }\right).$$

The simplified model described by Equation (8) was adopted to describe C45 steel, and the values of the model coefficients are given in Table 2.

The parameters of the simulation and experimental tests are presented in Table 3.

## 3. Results and Discussion

### 3.1. Numerical Simulations

**Figure 3:**
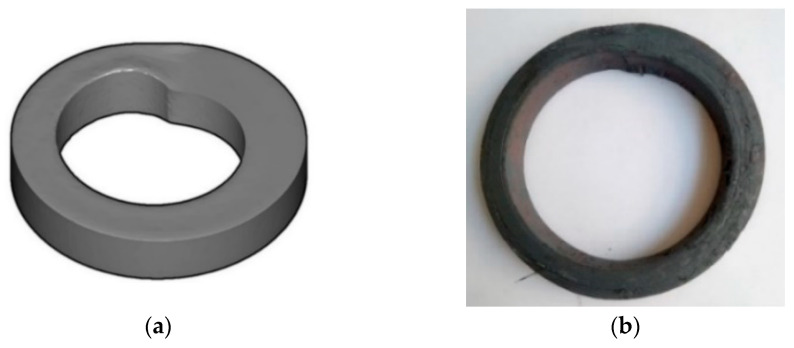
Defective rings obtained in a process carried out with a rotational speed of *n*_1_ = 60 rpm and the feed speed of the main roll *V*_1_ = 35 mm/s: (**a**) theoretical analysis and (**b**) experiment.

The first stage of the research was to verify the accuracy of the simulation. For this purpose, a series of calculations and experimental tests were performed on the rolling of a preform heated to a temperature of 1100 °C for various feed speeds of the main roll in the process. The results of the theoretical analysis disclosed that the maximum feed speed at which the correct course of the process is possible for the rotational speed of the main roll *n*_1_ = 60 rpm is 30 mm/s. For a feed speed of 35 mm/s, a defective ring was obtained (Figure 3a). The ring was deformed as a result of the slip between the tool and the product. The same results were obtained during experimental tests (Figure 3b). In this respect, a good correlation between theoretical and experimental results was established. The results confirmed the correct selection of the friction factor in the simulations which has a decisive impact on the occurrence of the slip phenomenon.

**Figure 4:**
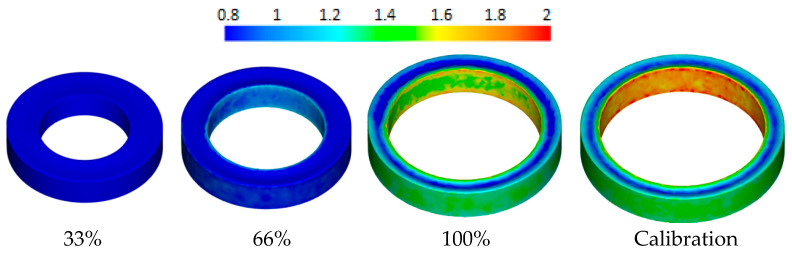

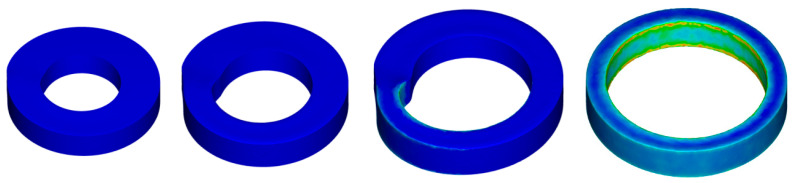
Results of numerical simulations of two extreme cases (5 mm/s—**upper row**, 35 mm/s—**lower row**) with distribution of equivalent plastic strain; temperature 1100 °C.

Subsequently, the shape and dimensions of the cross-section and the diameters of the rings obtained in the simulation and in the experiment were compared. Figure 4 shows the progression of the rolled ring shape for the lowest (5 mm/s) and the highest (30 mm/s) correct feed speed of the main roll. At a low feed speed value of the main roll (5 mm/s), the ring changes its geometry, and in particular, its cross-section, continuously—there is no visible recess of the tools in the formed product. The ring is deformed several times in the gap between the tools, and during one revolution, there is a slight reduction in the thickness of the forging. During the forging shaping process with the maximum feed speed of the main roll (30 mm/s), a large reduction in cross-section (tool recess) is visible. The ring makes a significantly smaller number of revolutions than when forming at a speed of 5 mm/s. Both processes are characterized by different characteristics of the change in the cross-section of the ring. At small values of the roll feed speed, during the calibration stage (after stopping the linear movement of the main roll, *V*_1_ = 0 mm/s), the formed element achieves its final profile without a significant change in the shape of the cross-section. In the case of high *V*_1_ feed speed, a noticeable change in the product geometry occurs during the calibration stage. This is due to the larger amount of material which is moved through the roll gap. The material in this stage flows mainly axially, increasing the height of the ring. Therefore, the ring has a smaller final diameter. The simulation results justify the conclusion that with the increase in the feed speed of the main roll, rings with greater widening are obtained.

**Figure 5:**
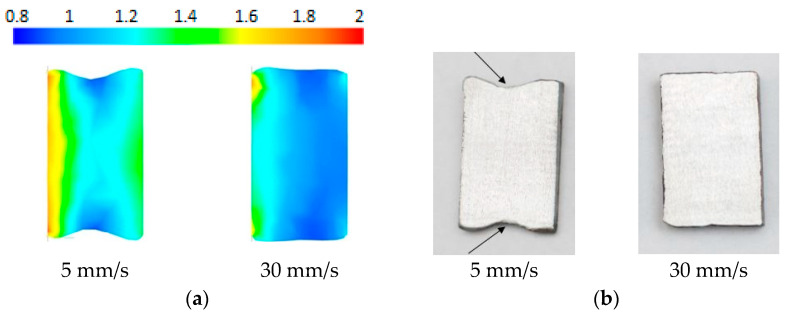
The shape of the cross-section of the ring obtained in simulation (**a**) and experimental tests (**b**) for the feed speed *V*_1_ of 5 mm/s and 30 mm/s and a preform temperature of 1100 °C; arrows indicate “fishtail” defect.

Figure 5a shows the distribution of equivalent plastic strain in the cross-section of rings rolled with feed speed *V*_1_ of 5 mm/s and 30 mm/s. It can be observed that the highest deformation values occur near the contact surface of the material with the tool, both on the side of the main roll and the mandrel. Increased local material flow increases the height of the ring cross-section in places of contact with the tools compared to the height in the middle of the cross-section width. The distribution of deformations in the cross-section is more uniform for the feed speed *V*_1_ = 30 mm/s (*k* = 0.025) which results in reduced deformation of the cross-section. As shown by the simulation results, the higher the feed speed of the main roll, the smaller the fishtail defect. In order to verify the theoretical results, experimental tests were performed for the same speed parameters. The actual cross-sections of the obtained rings are shown in Figure 5b.

The average height *h_fa_*, the external diameter *D_f,_* and internal diameter *d_f_* of the finished ring depending on the feed speed and the coefficient *k* determined by Equation (7) are shown in Table 4. The results indicated that the axial flow of the material is greater in the simulations than in the experiment. The theoretical height is significantly higher than the experimental height, therefore, the theoretical diameters are smaller than those obtained in the experiment. For each case, the percentage relative error *E* was calculated from the dependence:

(10) $$E=\frac{\left|W_{exp}-W_{theor}\right|}{W_{exp}}\cdot 100\;\%,$$

where:

*W_theor_*—*h_fa_*, *D_f_*, *d_f_* value obtained in the simulation,

*W_exp_*—*h_fa_*, *D_f_*, *d_f_* value obtained in the experiment.

Comparison of the numerical results with experimental results showed good qualitative correspondence (similar shapes of ring cross-sections) and reduced quantitative correspondence. Dimensional differences are relatively large, the percentage error is in the 5.64–10.24% range. Based on that, it was assumed that further research aimed at determining quantitative relationships would be performed experimentally.

### 3.2. Experimental Tests

In order to determine the influence of the process speed parameters and the preform temperature on the widening α and the distortion of the cross-section (*β* coefficient), experimental tests were carried out by heating the preform to temperatures of 900–1200 °C with steps of 100 °C and at *V*_1_ various feed speeds of the main roll (at constant rotational speed of the main roll *n*_1_ = 60 rpm). The *V*_1_ speed for each temperature was increased starting from 5 mm/s in 5 mm/s steps until the slip occurred. The temperature and speed parameters used in the experimental tests are presented in Table 5, in which the result of each test is also indicated (OK—the correct ring forging was obtained, S—the slip occurred). As depicted, the temperature of the preform has a significant impact on the occurrence of the slip; the slip occurred at the lower *V*_1_ speed with the growth in the temperature of the preform. The *α* and *β* coefficients were determined only for correct cases, therefore, the data range is different for different temperatures.

**Figure 6:**
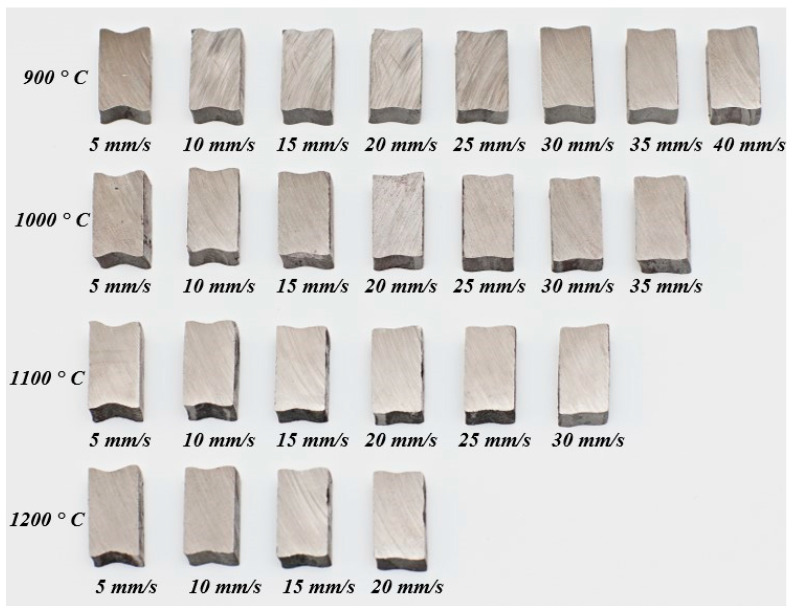
Cross-sections of the rings were obtained with 4 different initial temperatures of the preform at different feed speeds of the main roll *V*_1_ (*n*_1_ = 60 rpm).

The cross-sections of forgings obtained in experimental tests for individual temperature and speed conditions are shown in Figure 6. The outer diameter *D_f_* and internal diameter *d_f_* were measured in all rings (in four planes and the results were averaged) as well as the highest *h_fmax_* and the smallest *h_fmin_* cross-sectional height. The average cross-sectional height of the ring *h_fa_* was calculated according to Formula (4). Based on the measurements, the widening coefficient *α* and fishtail coefficient *β* were calculated.

**Figure 7:**
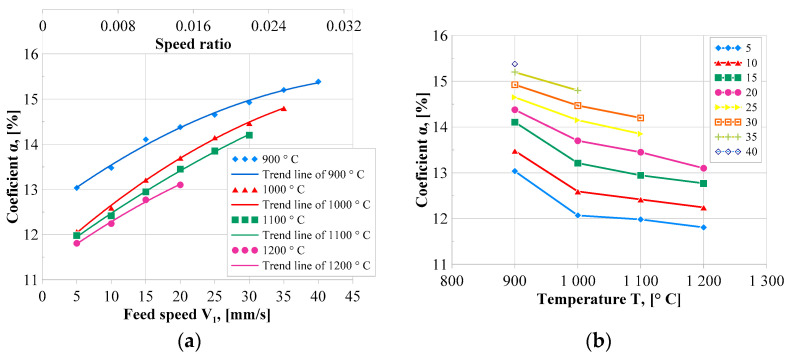
Coefficient *α* as a function of the *V*_1_ feed speed of the main roll and speed coefficient *k* (**a**) and coefficient *α* as a function of the initial temperature of the preform (**b**).

Figure 7a shows the correlation between the coefficients *α* and the *V*_1_ feed speed and the *k* speed ratio. The values of the *α* coefficient increase with the increase in the *V*_1_ feed speed and the *k* speed ratio. This means that with a higher ratio of the feed speed to the peripheral speed (rotational speed) of the main roll, the axial material flow increases, and the peripheral flow decreases. As a result, as the speed coefficient *k* increases, the internal and outer diameters of the produced rings decrease.

Figure 7b illustrates the dependence of the widening α on the heating temperature of the preform in the range of 900–1200 °C for different *V*_1_ speeds. The graph shows that the widening *α* decreases with the increase in the heating temperature of the preform. This means that the higher the heating temperature of the preform, the smaller the axial flow of the material, and thus the ring height is smaller, and the diameter is larger. The obtained interactions are important when designing the process because they contain information about the dimensions of the finished ring obtained with the selected rolling speed and temperature parameters.

**Figure 8:**
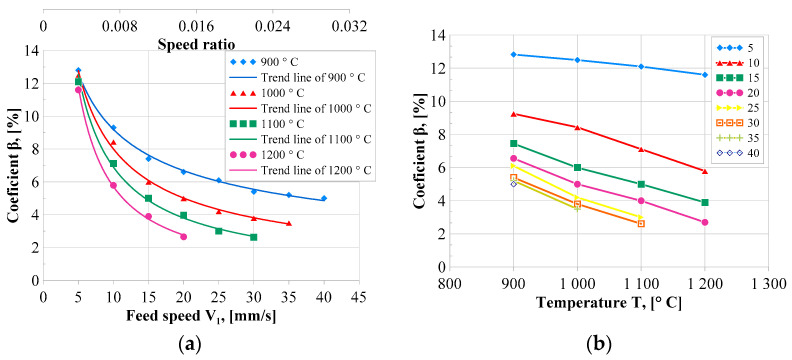
Coefficients *β* as a function of the *V*_1_ feed speed of the main roll and the speed coefficient *k* for various heating temperatures of the preform (**a**) and relationships between the *β* coefficient and the preform heating temperature for different values of the *V*_1_ feed speed (**b**).

Figure 8a shows the dependence of the fishtail coefficient *β* depending on the feed speed *V*_1_ of the main roll and the value of the coefficient *k*. The results proved that the coefficient *β* decreases exponentially with the increase of the feed speed *V*_1,_ and the speed coefficient *k*. This means that the higher the values of *V*_1_ and *k*, the more rectangular the shape of the cross-section becomes. This can be seen in Figure 6, which shows the cross-sections of the obtained rings.

The influence of temperature on the *β* coefficient (Figure 8b) generally demonstrates a decreasing trend, that is, the higher the temperature, the smaller the *β* coefficient. The intensity of this dependence devolves on the speed parameters. For the *V*_1_ = 5 mm/s, the influence of temperature is almost imperceptible, the *β* coefficient ranges from 11.6% to 12.8%. For larger values of the feed speed *V*_1_, the differences in the *β* coefficient are more pronounced and have smaller values for higher temperatures. Based on the results obtained, it can be concluded that in terms of cross-section distortion (fishtail defect), it is beneficial to heat the preform to higher temperatures.

### 3.3. Practical Aspects of Research Results

The research results allow the formulation of the general guidelines for designing the rolling process of C45 steel rings, in which *α* and *β* coefficients play an important role. It can be assumed that for other types, the determined relationships should be qualitatively similar, while quantitative relationships should be determined for a specific material type. For types of steel different than C45, the following set of rules steel should be treated as guidelines as to which process parameters are important and what their impact is on the process and the quality of the forging.

The first step in designing the rolling process is to determine the dimensions of the desired ring forging: final height *h_f_*, final external diameter *D_f_*_,_ and final internal diameter *d_f_*. These dimensions are defined based on the dimensions of the finished ring part increased by appropriate technological allowances which are removed by mechanical processing. Since in the radial rolling process, the cross-section of the forging is not a perfect rectangle, the height of the forging *h_f_* is equal to the average height of the forging *h_fa_*. It is proposed to calculate this height using the following formula:

(11) $$h_{fa}=h_{p}+h_{n},$$

where:

*h_p_*—height of the finished ring part (after lathing),

*h_n_*—machining allowance. 

It is suggested that the *h_n_* machining allowance meets the following requirement:

(12) $$h_{n}\geq h_{fa}\cdot \beta ,$$

where the coefficient *β* (in dimensionless form) is determined after selecting the preform temperature and speed parameters based on the test results presented in Figure 8.

In the next stage, the heating temperature of the preform, the rotational speed of the main roll, and the feed speed of the main roll (or mandrel) are determined.

The selection of the preform heating temperature depends on the criteria adopted. If high efficiency (high rolling speed) is important and/or minimization of the heating energy, lower temperatures ought to be used. However, if it is important to reduce the rolling force (for instance, not to overload the rolling mill) and/or to reduce material consumption (for instance, in the production of large series), it is more advantageous to use a higher preform heating temperature.

To prevent the slip effect, the ratio of the *V*_1_ feed speed to the peripheral speed (dependent on the rotational speed *n*_1_) of the main roll, that is, the coefficient *k* must not exceed the limit value, which can be described by the formula:

(13) $$k\leq k_{limit},$$

where: 

*k_limit_*—the limiting ratio of the feed speed to the peripheral speed of the main roll, determined in tests,

*k*—the ratio of the feed speed adopted for the process to the peripheral speed of the main roll, described by the relationship (7).

The above condition means that the *V*_1_ feed speed of the main roll cannot be too high in relation to its rotational speed *n*_1_. The values of the *k_limit_* coefficient for individual heating temperatures of the C45 steel preform in the radial rolling process are described in the study [23].

The selection of the feed speed and the rotational speed of the main roll depends on the criteria adopted. If the main criterion is to achieve the high process efficiency, it is beneficial to use the highest possible speeds for a given type of rolling mill, while observing the relationship (13). It must be noted that at high speeds the stability of the process is reduced and the rolled ring is more likely to be deformed or damaged. Therefore, if the main criterion is a smooth and repeatable process, appropriately lower speeds should be selected.

Subsequently, the dimensions of the preform must be established. The following order of selection of dimensions is suggested: (i) the selection of the *d*_0_ internal diameter (hole) of the preform, (ii) the selection of the *h*_0_ height of the preform, and (iii) the selection of the *D*_0_ outer diameter of the preform.

The *d*_0_ internal diameter of the preform ought to be as small as possible so that the energy consumption, material consumption, and tool wear are as low as possible in the process of forging the preform. However, the size of the opening is limited by the diameter of the mandrel, which must be of the appropriate stiffness. Based on own experience, it was estimated that in order to conveniently mount the preform on the mandrel, the internal diameter should be at least 4 mm larger than the mandrel diameter:

(14) $$d_{0}=d_{m}+4\;mm,$$

where:

*d*_0_—internal diameter of the preform, mm,

*d_m_*—mandrel diameter, mm (if the mandrel is stepped, the largest diameter should be used).

The *h*_0_ preform height should be lower than the average height of the finished forging by the value of the widening *α*, which depends on the process parameters (preform temperature and speed parameters). The preform height can be defined as:

(15) $$h_{0}=\frac{h_{fa}}{1+\alpha }\;,$$

where:

*h_fa_*—average height of the finished forging calculated based on the relationship (11),

*α*—widening coefficient, the value of which can be read from Figure 7 depending on the assumed preform temperature and speed parameters determined by the coefficient *k*.

The *D*_0_ outer diameter of the preform results from the principle of constant volume, that is, the equality of the volume of the finished ring forging and the volume of the preform excluding losses to forge scale, depending on the heating method used. It can be calculated using the equation:

(16) $$D_{0}=\sqrt{\frac{D_{f}^{2}-d_{f}^{2}}{h_{0}\cdot (1-\rho )}\cdot h_{fa}+d_{0}^{2}}$$

where:

*D_f_*, *d_f_* and *h_fa_*—outer diameter, internal diameter and average height of the finished forging,

*d*_0_, *h*_0_—internal diameter and height of the preform,

*ρ*—loss coefficient for forge scale depending on the method and time of heating.

## 4. Conclusions

The analysis of the above results allows us to conclude that the speed parameters and temperature of the formed material have a significant impact on the dimensions and shape of the ring cross-section and, consequently, on the outer diameter and internal diameter of rings rolled using the radial method. An understanding of these relationships is essential when designing the process of rolling rings with assumed final dimensions. Moreover, the trial and error phase is limited when starting the production of new ring forgings.

Based on the obtained research results, the following conclusions were formulated.

1. The share of the volume of material which flows axially and circumferentially during rolling depends on the process parameters, mainly the ring temperature and tool speed. Understanding the quantitative relationships between these parameters and the nature of material flow is the basis for the correct design of the process ensuring obtaining a forging with the assumed dimensions.
2. As the *V*_1_ feed speed of the main roll and the coefficient *k* increase, the widening of the cross-section of the rolled ring rises, that is the axial material flow is increased, while the peripheral flow is reduced. This means that the higher the *V*_1_ feed speed (coefficient *k*), the greater the height and smaller the diameter of the rolled ring.
3. The influence of the preform temperature on the widening (*α* coefficient) is inverse to the influence of the speed parameters. The higher the temperature of the preform, the smaller the widening.
4. A higher *V*_1_ feed speed of the main roll (and thus the coefficient *k*) causes distortion of the cross-section of the product in the form of an indent called a “fishtail”. Therefore, in order for the material losses resulting from the machining allowance to be smaller in the process of rolling rings with a rectangular cross-section, the speed parameters should be selected so that the coefficient *k* is as high as possible but it does not exceed the limit value.
5. The higher the temperature of the preform, the smaller the cross-section deformation (coefficient *β*).
6. Verification of the accuracy of calculations of the used program based on the finite element method showed that for the analyzed case, good compliance with the experimental results occurs in the qualitative analysis (ring shape), while there are discrepancies in the quantitative analysis (dimensional values). Considering this observation and the fact that most publications on the analysis of ring rolling processes are based on FEM analysis, it is recommended to use experimental research as the basic source of knowledge about the process.

## Figures and Tables

**Table 1:** Chemical composition of 1.0503 (C45) steel (wt%).

	C	Si	Mn	Cr	Ni	Mo	Cu	S	P	Ti	Fe
min	0.42	0.10	0.50	0.00	0.00	0.00	0.00	0.00	0.00	0.00	rest
max	0.50	0.40	0.80	0.30	0.30	0.10	0.30	0.045	0.045	0.05	

**Table 2:** Parameters of the Hansel–Spittel model for C45 steel.

Constans	*A*	*m*_1_	*m*_2_	*m*_3_	*m*_4_
Value	1521	0.00269	−0.12651	0.14542	−0.05957

**Table 3:** Parameters of experimental tests and numerical simulations.

Parameter	Value	Unit
Feed speed *V*_1_ of the main roll	5–45	mm/s
Rotational speed *n*_1_ of the main roll	60	rpm
Environment temperature	20	°C
Tools temperature	150	°C
Preform temperature	900–1200	°C
Main roll diameter	380	mm
Mandrel parameter	46	mm
Heat exchange coefficient beetwen tools and preform	10 000	W/(m^2^K)
Heat-air exchange coefficient	20	W/(m^2^K)
Friction factor *m*	0.74	-

**Table 4:** Comparison of the dimensions of the ring obtained in numerical simulation and in experimental tests.

Dimension	Temperature, °C	Feed Speed *V*_1_, mm/s	Theoretical Value, mm	Experimental Value, mm	Error *E*, %
*h_fa_*	1100	5	23.88	22.40	6.61
		30	25.15	22.84	10.11
*d_f_*		5	107.8	115.8	6.91
		30	101.7	113.3	10.24
*D_f_*		5	133.8	141.8	5.64
		30	127.7	139.3	8.33

**Table 5:** Parameters of experimental tests.

Feed Speed *V*_1_, mm/s	Temperature, °C			
	900	1000	1100	1200
5	OK	OK	OK	OK
10	OK	OK	OK	OK
15	OK	OK	OK	OK
20	OK	OK	OK	OK
25	OK	OK	OK	S
30	OK	OK	OK	S
35	OK	OK	S	S
40	OK	S	S	S
45	S	S	S	S

OK—the ring was forged correctly, S—the slip occurred.

## Data Availability

The data that support the findings of this study are available from the corresponding author, upon reasonable request.
